# Paraoxonase‐1 activity evaluation as a diagnostic and prognostic marker in horses and foals

**DOI:** 10.1111/jvim.15722

**Published:** 2020-03-10

**Authors:** Beatrice Ruggerone, Saverio Paltrinieri, Alessia Giordano, Donatella Scavone, Irene Nocera, Riccardo Rinnovati, Alessandro Spadari, Licia Scacco, Paola Pratelli, Micaela Sgorbini

**Affiliations:** ^1^ Department of Veterinary Medicine University of Milan Milano Italy; ^2^ Veterinary Teaching Hospital, University of Milan Lodi Italy; ^3^ Department of Veterinary Sciences University of Pisa Pisa Italy; ^4^ Veterinary Teaching Hospital, Department of Veterinary Sciences University of Pisa Pisa Italy; ^5^ Department of Veterinary Medical Sciences University of Bologna Bologna Italy; ^6^ Equivet Roma Hospital, Equine Veterinary Clinic Roma Italy; ^7^ Private Veterinary Practitioner Pisa Italy

**Keywords:** acute phase protein, inflammation, oxidative stress, prognosis, SIRS

## Abstract

**Background:**

In several species, paraoxonase‐1 (PON‐1) decreases during inflammation, because of the presence of oxidative stress; its measurement recently has been validated in horses, but its role as a clinical biomarker is unknown.

**Objectives:**

To evaluate sensitivity, specificity and likelihood ratio of PON‐1 activity to identify systemic inflammatory response syndrome (SIRS)‐positive horses or horses with a poor prognosis.

**Animals:**

One hundred seventy‐two blood samples from 58 sick horses from 3 different veterinary hospitals.

**Methods:**

In a cross‐sectional study, PON‐1 activity was measured upon admission and at 24‐hour intervals until discharge or death, and results were analyzed based on SIRS status and outcome.

**Results:**

No statistically significant difference was found in median PON‐1 activity between SIRS and non‐SIRS cases or between survivors and non‐survivors except for mares, in which PON‐1 activity was significantly lower in SIRS‐positive horses (*P* = .05). The sensitivity of PON‐1 activity in identifying horses with SIRS or negative outcome was low (0.0%‐46.2% depending on the examined group) but its specificity was high (87.0%‐100.0%). However, when PON‐1 is low, the likelihood of death is 2.40‐3.89 times higher than the likelihood of survival. Repeated measurement of PON‐1 after treatment does not predict outcome.

**Conclusions and Clinical Importance:**

Evaluation of PON‐1 activity in horses with inflammation might be advisable in the future, but only low activity at admission may be relevant in predicting SIRS or negative outcome.

## 1 INTRODUCTION

Paraoxonase‐1 (PON‐1) is an enzyme associated with high‐density lipoproteins (HDL) that protects low‐density lipoproteins (LDL) and HDL from peroxidation.^1^ It is mainly synthesized in the liver and transported in the plasma bound to HDL. Serum activity of PON‐1 decreases during inflammation. During the acute phase response, in both laboratory animals^2^, ^3^ and people,^4^ changes in HDL composition and structure inactivate the enzyme PON‐1 and hepatic gene expression of PON‐1 is inhibited.^2^, ^5^ For these reasons, PON‐1 is considered a negative acute phase protein (APP).^2^, ^5^

Few studies have investigated changes in PON‐1 activity in sick animals (cattle,^6^ cats,^7^ swine,^8^ and dogs^9^, ^10^). These studies also supported the role of PON‐1 as a negative APP in animals, but decreases of PON‐1 appear to be relevant only when inflammation is particularly severe and potentially associated with oxidative phenomena. Activity of PON‐1 can be evaluated using different substrates, but the paraoxon‐based method to measure serum PON‐1 activity has been validated only in dogs,^9^ cattle,^11^ and horses.^12^

Recently, the term systemic inflammatory response syndrome (SIRS), rather than endotoxemia, has been suggested to describe the clinical status of endotoxemic horses. Diseases that have been associated with SIRS in adult horses include especially those involving the gastrointestinal tract, such as the inflammatory intestinal diseases and strangulating obstructions.^13^

An early diagnosis should be the goal in management of SIRS patients, allowing starting adequate treatment in an early stage.^14^, ^15^

Our aim was to compare PON‐1 activity in sick horses classified as SIRS‐positive or SIRS‐negative, as proposed by others,^16^ and to investigate the performances of PON‐1 activity in terms of sensitivity, specificity, and likelihood ratio in identifying SIRS‐positive and negative horses. Finally, the possible prognostic role of PON‐1 activity was assessed by evaluating PON‐1 activity in sick horses based on clinical outcome.

## 2 MATERIALS AND METHODS

### 2.1 Samples and study design

The study was performed on 172 blood samples from 58 horses (36 mares, 17 geldings, and 5 stallions). Sick animals were referred to 3 different hospitals providing secondary health care.

Horses were classified as sick on the basis of clinical examination and ancillary tests (routine serum biochemistry and hematology, radiographs, ultrasound examination, and cytological and bacteriological evaluation of synovial fluid or bronchoalveolar lavage fluid).

The following data were recorded in order to classify and divide the sick horses in SIRS‐positive or SIRS‐negative groups^16^: presence of abnormal leukocyte count as leukopenia or leukocytosis (reference interval [RI], 5.0‐12.5 × 10^3^/μL), left shift (RI, >10% band neutrophils), hyperthermia or hypothermia (RI, 37.0°C‐38.5°C), tachycardia (RI, >52 beats per minute [bpm]), and tachypnea (RI, >20 breaths per minute). Horses with 0 or 1 abnormal criterion were included in the SIRS‐negative group, whereas horses with ≥2 abnormal criteria were included in the SIRS‐positive group.^16^

Retrospectively, sick horses also were divided into survivors and non‐survivors. Animals were considered survivors if they were discharged from the hospital, whereas they were considered non‐survivors if they died or were humanely euthanized because of severe medical prognosis rather than for economic reasons.

Although all the sick horses included in the study were admitted to the hospitals within 30 hours after the onset of the disease, it was not possible to standardize the time elapsed from the onset of clinical signs and the first sampling or to collect a complete history regarding the pre‐admission period. After the first sampling (T0), all of the horses received appropriate treatment based on the actual diagnosis and clinical presentation.

Blood samples were collected by the investigators within 1 hour after admission at the hospital, and additional samples then were collected at 24‐hour intervals until discharge or death or for a maximum of 96 hours. Lipemic samples were excluded from the study.

Blood samples were collected from the jugular vein for the determination of CBC and PON‐1 activity using a sterile syringe and 16G needle. Each blood sample was divided in 2 aliquots: a 1‐mL aliquot was collected in potassium ethylene diamine tetra‐acetic (K_2_EDTA) and analyzed using a cell counter (ProCyte Dx, IDEXX, Westbrook, Maine) within 5 minutes after collection. A second 2.5‐mL aliquot was collected in plain tubes and centrifuged at 2100 relative centrifugal force for 10 minutes within 4 hours of collection. The harvested serum was placed in sterile tubes, frozen at −20°C to be transported to the laboratory, where serum PON‐1 activity was measured in a single batch as described below.

The study was performed during the regular course of hospitalization of the animals and with the owner's written consent.

### 2.2 Measurement of serum PON‐1 activity

Serum PON‐1 activity was measured spectrophotometrically using an automated analyzer (Cobas Mira, Roche diagnostic, Basel, Switzerland), and an enzymatic method already validated in horses.^12^ Briefly, 6 μL samples were incubated at 37°C with 89 μL of distilled water and 100 μL of reaction buffer (glycine buffer 0.05 mM, pH 10.5 containing 1 mM of paraoxon‐ethyl, purity >90% [Sigma‐Aldrich, Saint Louis, Missouri], and 1 mM of CaCl_2_). The rate of hydrolysis of paraoxon to p‐nitrophenol was measured by monitoring the increase in absorbance at 504 nm using a molar extinction coefficient of 18.050 L/mol/cm^−1^ as previously suggested.^2^ The unit of PON‐1 activity expressed as U/mL is defined as 1 nmol of p‐nitrophenol formed per minute under the assay conditions.

### 2.3 Statistical analysis

Statistical analysis was performed using an Excel spreadsheet and specific software (Analyse‐it, Analyse‐it Software Ltd, Leeds, UK).

Results for PON‐1 activity upon admission to the hospital obtained from SIRS‐positive horses^16^ were compared with results from the SIRS‐negative horses, using the Mann‐Withney *U* test. The same test was used to compare the results obtained upon admission between survivors versus non‐survivors. A Fisher exact test was used to verify the association between categorized PON‐1 results (within versus below the RI) and SIRS‐positive or negative classification or outcome (survivors versus non‐survivors). Statistical differences were set for *P* < .05.

To assess diagnostic performance of PON‐1 activity in detecting SIRS‐positive horses, the numbers of samples from SIRS‐positive and SIRS‐negative subjects that upon admission had PON‐1 activity within or below the RI established in the previous study^12^ were counted. Data then were classified as follows:

- True‐positive (TP): Sick horses with PON‐1 activity lower than the RI and SIRS‐positive;
- False‐positive (FP): Sick horses with PON‐1 activity lower than the RI and SIRS‐negative;
- True‐negative (TN): Sick horses with PON‐1 activity within the RI and SIRS‐negative; and
- False‐negative (FN): Sick horses with PON‐1 activity within the RI and SIRS‐positive.

The same classification scheme was used to assess the diagnostic performance of PON‐1 activity in detecting animals with a poor prognosis, considering as “positive” the non‐survivors and as “negative” the survivors.

In both instances, TP, TN, FP, and FN results were used to calculate sensitivity, specificity and likelihood ratios using standard formulas.^17^, ^18^

Results from sequential samplings collected after treatment were not statistically compared to each other because of the low number of samples that had comparable follow‐up. Hence, the analysis of results collected during the follow‐up was limited to a visual observation of the trend recorded in animals that survived or died despite treatment.

## 3 RESULTS

### 3.1 Caseload

The 58 sick horses were grouped as follows on the basis of the final diagnosis: obstructive or strangulated colic (n = 34), other gastrointestinal diseases (n = 4:3/4, enteritis; 1/4, esophageal obstruction), bacterial pneumonia or pleuropneumonia (n = 4), trauma (musculoskeletal or skin or both; n = 8), neoplasia (n = 2), and paraphimosis, neurological signs, pyometra, sinusitis, pericarditis and Cesarean section (n = 1 each).

The SIRS‐positive horses consisted of 35/58, whereas 23/58 were classified as SIRS‐negative. The SIRS‐negative horses had obstructive (7/23) or strangulated (4/23) colic, trauma (4/23 horses: 1/4, mild fracture; 3/4, wound), 2/23 neoplasia and granulomatous enteritis, respectively, and 1/23 paraphimosis, pyometra, sinusitis, and choke, respectively. The SIRS‐positive horses had obstructive (4/35) and strangulated (19/35) colic, 4/35 pneumonia or pleuropneumonia (3/4, pneumonia; 1/4, pleuropneumonia), 3/35 severe trauma, 1/35 pericarditis, Cesarean section, and granulomatous enteritis associated with dermatitis, respectively.

The SIRS score was 4 in 3/35 horses, all affected by pneumonia or pleuropneumonia, 3 in 15/35 animals (1/15 obstructive and 10/15 strangulated colic; other gastrointestinal disease, 1/15; trauma, 2/15; pneumonia, 1/15), and 2 in 17/35 (3/17 obstructive and 9/17 strangulated colic; trauma, 3/17; 1/17, pericarditis and Cesarean section, respectively).

Eighteen of 58 horses died despite treatment. Nine of these 18 horses were euthanized because of worsening clinical condition and not for economic reasons and 9/18 died spontaneously. Non‐survivor horses were affected by obstructive (n = 3) or strangulated colic (n = 9), pneumonia or pleuropneumonia (n = 4), trauma and other gastrointestinal diseases (n = 1 each). Thirteen horses died or were euthanized after the first sampling, 1 after the third sample, and 4 after the fifth and last sample.

### 3.2 Comparison of results recorded before treatment

At admission, 11/58 horses had PON‐1 activity lower than the lower limit of the RI of adult horses (38.0 U/mL). However, when results recorded in males, females or geldings were compared with the lower limit of the specific RI (males, 38.4 U/mL; females, 37.3 U/mL; geldings, 33.2 U/mL), only 10 horses (9 females and 1 gelding) had low PON‐1 activity.

No differences were found in PON‐1 activity between SIRS‐negative (mean ± SD, 49.1 ± 8.2 U/mL; median, 46.5 U/mL; I‐III interquartile range, 43.6‐54.3 U/mL) versus SIRS‐positive horses (45.3 ± 11.9 U/mL; 45.8 U/mL; 35.4‐54.6 U/mL; *P* = .21) and between survivors (47.9 ± 9.1 U/mL; 46.5 U/mL; 39.9‐54.8 U/mL) versus non‐survivors (44.4 ± 13.5 U/mL; 44.1 U/mL; 33.3‐53.4 U/mL; *P* = .27). Contingency analysis showed a significant association between categorized PON‐1 activity and SIRS classification (positive versus negative; *P* = .002) and between categorized PON‐1 activity and outcome (*P* = .02).

No significant differences were found when the analysis was restricted to geldings (SIRS‐negative: 46.2 ± 6.4 U/mL; 44.6 U/mL; 42.9‐50.4 U/mL; n = 11; versus SIRS‐positive: 44.9 ± 15.7 U/mL; 44.4 U/mL; 30.7‐54.4 U/mL; n = 6; *P* = .81; survivors: 44.5 ± 7.7 U/mL; 43.8 U/mL; 38.9‐49.2 U/mL; n = 12; versus non‐survivors: 48.9 ± 15.1 U/mL; 50.0 U/mL; 38.8‐58.5 U/mL; n = 5; *P* = .38). Conversely, in mares, PON‐1 activity was significantly lower (*P* = .05) in SIRS‐positive horses (44.8 ± 11.2 U/mL; 45.8 U/mL; 35.4‐54.5 U/mL; n = 27) compared with SIRS‐negative horses (53.9 ± 9.1 U/mL; 54.6 U/mL; 48.9‐56.9 U/mL; n = 9).

**Figure 1:**
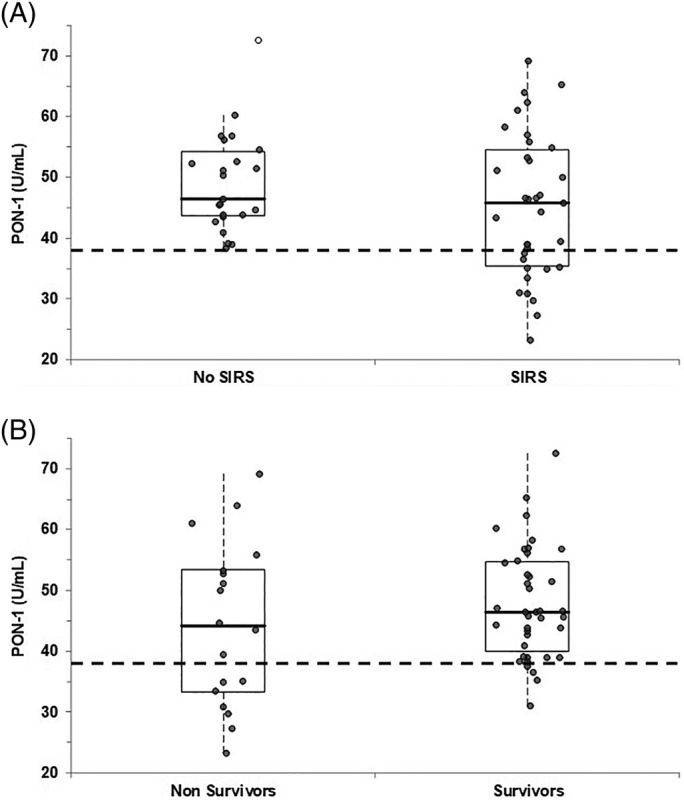
A, Distribution of results according to the systemic inflammatory response syndrome (SIRS) status. B, Distribution of results according to the outcome. The boxes indicate the I‐III interquartile range (IQR), the horizontal black line indicate the median values, whiskers extend to further observation within quartile I minus 1.5 × IQR or to further observation within quartile III plus 1.5 × IQR. The white circles indicate the outliers. The dotted line indicates the lower limit of the RI reported in healthy horses

As noted in Figure 1A, horses with PON‐1 activity lower than the RI were found only in the SIRS‐positive group, that, however, also included several horses with normal PON‐1 activity. Conversely, either horses with low PON‐1 activity or horses with normal PON‐1 activity were found in both groups on the basis of outcome (Figure 1B). The number of FP, FN, TP, and TN recorded in each group, and specificity, sensitivity or positive likelihood ratio calculated are reported in Table 1.

**Table 1:** Sensitivity (Sens), specificity (Spec), and positive likelihood ratio (LR+) in the examined subpopulation according to the SIRS status and the outcome

		TP	FN	FP	TN	TOT	Sens (%)	Spec (%)	LR+
SIRS	Whole caseload	10	25	0	23	58	28.6 (16.3‐45.1)	100.0 (85.7‐100.0)	n.c.
	Stallions	0	2	0	3	5	0.0 (0.0‐65.8)	100.0 (43.9‐100.0)	n.c.
	Mares	8	19	0	9	36	29.6 (19.5‐48.5)	100.0 (70.1‐100.0)	n.c.
	Geldings	2	4	0	11	17	33.3 (9.7‐70.0)	100.0 (74.1‐100.0)	n.c.
Outcome	Whole caseload	7	11	4	36	58	38.9 (20.3‐63.4)	90.0 (76.9‐98.0)	3.89
	Stallions	0	0	0	5	5	n.c.	100.0 (56.6‐100.0)	n.c.
	Mares	6	7	3	20	36	46.2 (23.2‐70.9)	87.0 (67.9‐95.5)	3.54
	Geldings	1	4	1	11	17	20.0 (3.6‐62.4)	91.7 (64.6‐98.5)	2.40

*Note*: The 95% confidence intervals of Sens, Spec, is reported in brackets.

Abbreviations: FN, false negative; FP, false positive; n.c., not computable; SIRS, systemic inflammatory response syndrome; TN, true negative; TOT, total number; TP, true positive.

As shown in Table 1, low PON‐1 activity has an absolute specificity for the diagnosis of SIRS (no FP were found, independent of group and corresponding cutoff). Conversely, PON‐1 activity has a variably low sensitivity, because the number of FN (normal PON‐1 activity in SIRS‐positive horses) was high, especially in male adults.

When animals are grouped based on outcome, specificity, although high, is not absolute, and FP results may occur. However, when PON‐1 activity is low, the likelihood to have a poor prognosis is 2.40 to 3.89 times higher than the likelihood to have a positive response to treatment.

### 3.3 Results recorded during the follow‐up

Results recorded in the 36 horses repeatedly sampled during follow‐up are reported in Supplementary Table S1. Sampling was repeated in 5 horses that had normal PON‐1 activity upon admission but died despite treatment (non‐survivors). Analysis of sequential samplings indicated that none of these horses had persistent decreases of PON‐1 activity over time: only 1 of these horses had low PON‐1 activity before death, whereas 1 had a transient decrease in PON‐1 activity 24 or 48 hours after treatment, but PON‐1 activity returned to within normal limits at the last 2 samplings. However, a transient decrease followed by rapid normalization also was observed in 6 of the 28 survivors, which had normal values upon admission and were sampled at least for 48 hours.

Only 3 survivors that had low PON‐1 activity upon admission were repeatedly sampled during follow‐up. In all 3 horses, activity increased over time and in 2 of them PON‐1 activity rapidly returned to within normal limits.

## 4 DISCUSSION

In several species, the antioxidant enzyme PON‐1 decreases in association with oxidative stress that characterizes sepsis.^5^, ^6^, ^7^, ^8^, ^9^, ^10^ Our study was designed to assess whether the activity of the antioxidant enzyme PON‐1 may serve as a diagnostic or prognostic marker in horses. To this aim, a paraoxon‐based method recently validated in horses^12^ was used.

However, our results only partly support a possible role for PON‐1 as a diagnostic marker in horses, because low PON‐1 activity may be associated with positive SIRS status, whereas normal PON‐1 activity does not rule out negative SIRS status.

All of the horses with low PON‐1 activity upon admission were also SIRS‐positive, whereas several SIRS‐positive horses had normal PON‐1 activity. Moreover, the horses with poor prognosis included either horses with low PON‐1 activity or horses with normal PON‐1 activity.

Hence, PON‐1 activity may be a good marker of SIRS in horses only when results recorded upon admission are low, as opposed to what has been reported in other species.^10^, ^11^ This difference may suggest that, compared with other species, horses have different PON‐1 metabolism, or less severe oxidative stress during inflammation. Different from other inflammatory markers, PON‐1 activity decreases only if oxidative stress is present.^2^, ^5^ Future studies including measurement of other markers of oxidation (eg, thiobarbituric acid substances, reactive oxygen species) may allow clarification. The hypothesis that not all inflammatory conditions are associated with oxidative stress is supported by results of previous studies in dogs that found decreased PON‐1 activity only in some, but not all, dogs with increases in other APP.^9^, ^10^ Moreover, the horses included in our study were affected by different diseases which likely have different severity of oxidation, which could have contributed to the variability of the results. Although most horses were affected by colic, the study population was heterogeneous in terms of type of disease, the pathogenesis of which may or may not have included oxidative phenomena. Unfortunately, the number of cases in the different disease categories was not homogeneous and a reliable statistical comparison of results obtained in the different groups was not possible. Additionally, it was not possible to standardize the time elapsed between the onset of inflammation and the first sampling, and therefore the magnitude of inflammation may be different in horses examined just after the onset of clinical signs or a few hours later. In people, sepsis is the condition mostly associated with oxidation.^1^ The lack of differences of PON‐1 activity between SIRS‐negative and SIRS‐positive horses may be related to the fact that SIRS may or may not be associated with sepsis. Thus, it might be possible that SIRS‐positive horses actually do not have systemic spread of the septic process and vice versa. Future studies based on other markers of systemic inflammation such as serum amyloid A, that in horses acts as a major APP,^19^ or based on reliable tests to definitely classify the SIRS‐positive horses as septic or non‐septic would be useful to more accurately classify horses or foals with SIRS.

In other species, decreased PON‐1 activity is a negative prognostic marker and may predict outcome.^10^, ^11^, ^20^ This does not seem to be true in horses, because the specificity of decreased PON‐1 was not absolute, possibly because of the same factors described above. However, comparison of results from horses with positive or negative outcome also may have been biased by the low number of observations, on one hand, and by the wide distribution of results in the non‐survivors on the other hand. It is possible that, with increasing numbers of horses with a negative prognosis, these differences would become significant.

Independent of the mechanisms responsible for the lack of decreases in PON‐1 activity, the fact that all of the horses with low PON‐1 activity also were positive for SIRS may have some practical utility. In routine practice, when PON‐1 activity upon admission is lower than the RI, according to our data, SIRS is always present, as demonstrated also by the Fisher test, and the likelihood for the horse to not survive is 2.40 to 3.89 times higher than the likelihood to respond to treatment, supporting the hypothesis that PON‐1 activity may provide useful information in clinical practice. Conversely, normal PON‐1 activity does not exclude the presence of SIRS or a possible negative prognosis despite treatment during follow‐up. Therefore, it would be advisable to measure PON‐1 activity upon admission and pay particular attention to the management of horses with low PON‐1 activity.

Additionally, our study failed to provide information on the possible utility of sequential measurement of PON‐1 activity after administration of treatment to achieve prognostic information. The number of horses that died and were repeatedly sampled was too low to draw any conclusions, as was the number of horses that had low PON‐1 activity upon admission and survived. Some of the horses with normal PON‐1 activity at admission that died during follow‐up had transient decreases of PON‐1 activity below the lower limit of the RI, but this also happened in some of the survivors. In other species, a rapid increase of PON‐1 activity was recorded in animals that had abnormal activity at admission and responded to treatment.^9^ Therefore, it would be advisable in the future to increase the number of animals, and especially the number of non‐survivors with repeated samplings.

Finally, although a previous study^12^ demonstrated that the RI varies among horses of different breeds or use, we did not have the opportunity to apply breed‐related RIs because the number of horses with low PON‐1 activity per breed was too low to allow a reliable statistical comparison. Therefore, it also may be advisable to investigate, by analyzing a larger caseload, whether diagnostic performance could improve by comparing the results of sick horses with breed‐specific RIs.

In conclusion, our study suggests that horses with low PON‐1 activity likely have SIRS and may have a negative prognosis, whereas normal PON‐1 activity does not allow exclusion of these 2 considerations. This may be a consequence of different metabolism of paraoxonase‐1 in horses, lower magnitude of oxidation associated with SIRS in this species or the sensitivity of the assessment of SIRS status in identifying SIRS‐positive horses. The exact mechanism responsible for this difference should be investigated in future studies based on a higher number of horses, on a higher number of horses sampled repeatedly during follow‐up, and on a wider panel of tests that should include other markers of oxidation or systemic inflammation and horses with experimentally induced SIRS, by which several variables typical of field studies may be standardized.

## CONFLICT OF INTEREST DECLARATION

Authors declare no conflict of interest.

## OFF‐LABEL ANTIMICROBIAL DECLARATION

Authors declare no off‐label use of antimicrobials.

## INSTITUTIONAL ANIMAL CARE AND USE COMMITTEE (IACUC) OR OTHER APPROVAL DECLARATION

Approval of the Ethical committee of the University of Pisa (prot. n. 23 506/16).

## HUMAN ETHICS APPROVAL DECLARATION

Authors declare human ethics approval was not needed for this study.

## Supporting information


**Appendix** **S1**: Supporting Information
