# Supplementary material for: Paraoxonase‐1 activity evaluation as a diagnostic and prognostic marker in horses and foals
Source: J Vet Intern Med. 2020 Mar 10;34(2):949–54. doi: 10.1111/jvim.15722 (PMC7096640; doi:10.1111/jvim.15722)
Supplement: Supplementary file 1 — Appendix S1: Supporting Information [file JVIM-34-949-s001.pdf]

Supplementary Table 1

Results of PON-1 activity (U/mL) recorded in sequential samplings of the 36 horses that were survivors (S) or non-survivors (NS) at the end of the study period and that were sampled during the follow up. Results below the lower reference limit are reported in bold.

#	Outcome	**T0**	**T24**	**T48**	**T72**	**T96**		#	Outcome	**T0**	**T24**	**T48**	**T72**	**T96**
9	S	**35.2**	**32.5**	**36.5**	**37.3**			36	S	50.4	**35.3**	46.1		
10	S	**36.6**	41.7	54.5	50.1			38	S	51.2	46.3	53.4	52.4	
11	S	**37.5**	46.4	56.9	51.9	49.1		39	S	51.5	47.6			
12	S	38.3	44.2	40.2	42.8			40	S	52.3	64.4	60.6	61.5	62.1
15	S	38.9	**30.5**	39.5	37.7	43.6		41	S	52.6	45.8	58.7	40.5	42.8
17	S	39.2	49.5					43	NS	53.2	39.4	**30.8**	38.3	43.1
20	S	42.7	45.9	46.1				44	S	54.6	63.8	60.2	56.9	
21	S	43.3	**34.0**	43.1	42.5	42.9		45	S	54.9	52.4	54.7	58.3	
22	NS	43.6	46.7	44.5				46	NS	55.9	44.9	43.6	**37.5**	**36.7**
23	S	43.8	43.8	45.5				48	S	56.9	45.9	47.3	55.3	
24	S	43.8	40.6					50	S	57.0	57.0	60.4		
25	S	44.4	**37.4**	**34.0**	**37.0**	38.4		51	S	58.3	50.9	63.4	58.3	58.3
27	S	45.4	**37.6**	**35.5**	40.6	39.2		52	S	60.3	43.1	49.6	49.0	45.5
28	S	45.6	49.4	52.3	55.3			53	NS	61.0	62.6	48.1	45.4	59.9
29	S	45.8	51.5					54	S	62.3	42.7	33.6	38.4	45.0
31	S	46.5	46.9	49.1	47.4	53.4		55	NS	64.0	45.1	52.5	53.1	51.9
32	S	46.6	**37.0**	**33.9**	38.4	50.9		56	S	65.3	59.2	59.7	48.8	63.0
34	S	47.1	39.4	44.6	42.4	38.3		58	S	72.6	57.2	68.3	65.7	54.5
